# Risk of Sarcopenia and Osteoporosis in Male Tuberculosis Survivors: Korea National Health and Nutrition Examination Survey

**DOI:** 10.1038/s41598-017-12419-y

**Published:** 2017-10-13

**Authors:** Chang-Jin Choi, Whan-Seok Choi, Churl-Min Kim, Sook-Young Lee, Kyung-Soo Kim

**Affiliations:** 1 Department of Family Medicine, Seoul St. Mary’s Hospital, College of Medicine, The Catholic University of Korea, Seoul, Republic of Korea; 2 Division of Pulmonology Medicine, Department of Internal Medicine, Seoul St. Mary’s Hospital, College of Medicine, The Catholic University of Korea, Seoul, Republic of Korea

## Abstract

Short-term prospective studies have suggested that pulmonary tuberculosis (TB) preludes permanent loss of lean tissue and fat mass even when TB treatment is effective. The aim of this study was to estimate the risk of sarcopenia and osteoporosis among Korean male TB survivors. Data of the population-based, Korea National Health and Nutrition Examination Survey (KNHANES) (2008–2011) were analyzed, including 3,228 males aged 50 years or older who underwent chest X-ray (CXR) and dual-energy x-ray absorptiometry (DEXA). TB survivors having both medical history and TB scars on CXR had increased risk of sarcopenia (odds ratio [OR] 3.44, 95% confidence interval [CI] 1.79–6.68) and osteoporosis (OR 1.75, 95% CI 1.04–2.95) after adjusting for age, height, smoking, alcohol, physical activity, serum 25-hydroxyvitamin D, parathyroid hormone level, education, and fat mass index. Having TB scars on CXR without medical history of TB was an independent risk factor of sarcopenia (OR 2.05, 95% CI 1.05–4.00), but not a risk factor of osteoporosis. Sarcopenia and low bone mineral density are prevalent in pulmonary TB survivors with TB scars on CXR. Medical history of TB with TB scars on CXR is an independent risk factor for sarcopenia and osteoporosis.

## Introduction

Tuberculosis (TB) is the world’s deadliest infectious disease. In 2015, more than 10.4 million people became ill with TB and 1.4 million died, with an estimated 4.3 million not diagnosed or reported^1^. TB is a major public health problem in South Korea. The prevalence of active TB in Korea was 5,065/100,000 population in 1965^2^. It has rapidly decreased since then. The estimated incidence in 2015 was 80 per 100,000 population, corresponding to an intermediate national burden of TB^1^.

Nutritional status which is usually determined by body mass index (BMI) influences the clinical presentation, clinical course, and outcome of TB infection. Under-nutrition is a risk factor of active TB infection^3^. It is a determinant of severity^4^ and relapse of TB^5^. Lower BMI is associated with poorer treatment response^6,7^ and higher risk of mortality during TB treatment^8^. Under-nutrition is both an important risk factor and a common consequence of active TB infection. Weight loss and anorexia are typical presenting symptoms of active TB^9^. Most patients gain weight during treatment. However, after treatment has ended, they continue to remain underweight compared to healthy controls^10^. In prospective cohort studies with a follow-up of 6 months to 2 years, TB patients with wasting have failed to recover to the same levels of body weight as those without wasting^11–13^. These results suggest that full nutritional recovery takes much longer than effective TB treatment. Therefore, TB might lead to permanent loss of lean tissue and fat mass. The high risk of pathologic loss of lean tissue in TB survivors can increase future health risks, including sarcopenia and low bone mineral density (BMD).

Previous studies on changes in body composition after treatment of TB were short-term studies using anthropometric measurements or bioelectrical impedance analysis. The gold-standard of body composition analysis is dual-energy x-ray absorptiometry (DEXA). A bioelectrical impedance analysis prediction method has not been fully validated. To the best of our knowledge, no previous studies have reported the long-term consequence of body composition and BMD for pulmonary TB survivors using DEXA.

Therefore, the objectives this study were: 1) to compare body composition and BMD of participants based on different evidence of prior TB (i.e., medical history or radiologic evidence) and 2) to compare the risk of sarcopenia and osteoporosis of pulmonary TB survivors to those without evidence of TB using male data from a representative Korean national population study.

## Results

### Participants

**Figure 1:**
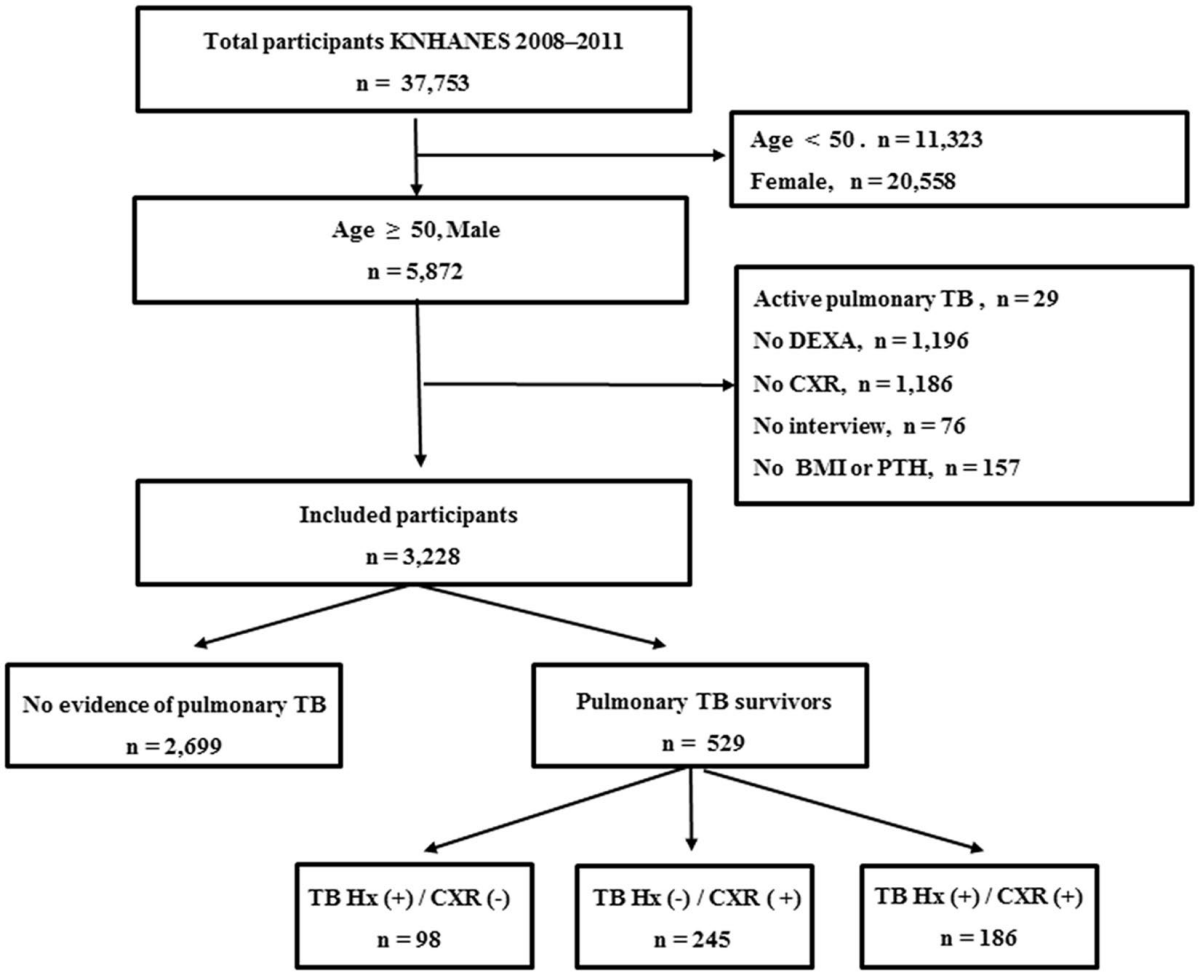
Flowchart of the study population. BMI, body mass index; CXR, chest x-ray; DEXA, dual-energy x-ray absorptiometry; Hx, history of physician diagnosis; PTH, parathyroid hormone; TB, tuberculosis; Hx (+)/CXR (−), subjects with a history of TB without TB scars on CXR; Hx (−)/CXR (+), subjects with TB scars on CXR without a history of TB; Hx (+)/CXR (+), subjects having both a history of TB and TB scars on CXR.

Among 3,228 male subjects aged 50 years or older, 529 (16.4%) were TB survivors. Of these, 98 (3.0%) only had a history of TB, 245 (7.6%) only had radiographic evidence of TB on chest X-ray (CXR), and 186 (5.8%) had both a history and CXR evidence of TB (Fig. 1). Participants having pulmonary TB scars on CXR (with or without a history of TB) were more likely to be older with lower BMI, lower educational background, and less frequent alcohol consumption than those without TB scars on CXR. Subjects with a history of TB without TB scars were demographically similar to those without evidence of TB (Table 1).

**Table 1:** Baseline characteristics of study participants (n = 3,228).

	No evidence of pulmonary TB	TB survivors			
		Hx (+)/CXR (−)	Hx (−)/CXR (+)	Hx (+)/CXR (+)	*P*
	n = 2,699	n = 98	n = 245	n = 186	
**Age, years**					
50–<60	1136 (57.1)	38 (54.7)	47 (29.2)	50 (34.6)	<0.001
60–<70	966 (28.4)	37 (32.1)	85 (32.7)	67 (35.6)	
≥70	597 (14.6)	23 (13.2)	113 (38.1)	69 (29.8)	
Mean	59.84 ± 0.20	60.40 ± 0.98	65.72 ± 0.74	64.66 ± 0.72	<0.001
Height, cm	167.27 ± 0.15	168.76 ± 0.84	165.89 ± 0.44	166.90 ± 0.56	0.005
Weight, kg	67.62 ± 0.25	68.00 ± 1.23	62.37 ± 0.79	61.69 ± 0.87	<0.001
**BMI, kg/m** ^**2**^					
<18.5	61 (1.7)	6 (3.5)	25 (9.6)	16 (8.4)	<0.001
18.5–<23	923 (31.8)	33 (31.6)	115 (48.0)	103 (57.2)	
23–<25	776 (29.0)	24 (25.4)	46 (19.3)	40 (18.0)	
≥25	939 (37.5)	35 (39.5)	59 (23.1)	27 (16.5)	
Mean	24.13 ± 0.07	23.82 ± 0.35	22.62 ± 0.25	22.10 ± 0.26	<0.001
**Smoking**					
Never	450 (16.0)	20 (17.4)	32 (10.9)	28 (13.5)	0.097
Ex-smoker	1312 (46.3)	56 (56.0)	129 (48.9)	108 (56.5)	
Current	937 (37.7)	22 (26.6)	84 (40.2)	50 (30.0)	
Pack/year	23.71 ± 0.50	22.18 ± 2.65	27.03 ± 1.78	25.40 ± 1.80	0.215
Physical activity*					
No	1796 (65.5)	59 (54.0)	173 (70.5)	132 (73.1)	0.040
Yes	903 (34.5)	39 (46.0)	72 (29.5)	54 (26.9)	
**Alcohol** ^**†**^					
No	834 (28.4)	25 (21.7)	101 (37.9)	63 (33.8)	0.014
Yes	1865 (71.6)	73 (78.3)	144 (62.1)	123 (66.2)	
**Education**					
<Elementary	909 (29.7)	26 (27.3)	112 (41.7)	75 (40.2)	0.010
Middle school	562 (21.3)	23 (19.4)	46 (20.7)	48 (26.5)	
High school	734 (29.4)	27 (29.9)	56 (24.0)	38 (18.9)	
≥College	494 (19.7)	22 (23.5)	31 (13.6)	25 (14.3)	
**Income**					
lowest	640 (23.6)	27 (23.7)	58 (24.0)	45 (23.9)	0.939
lower middle	677 (25.2)	25 (27.0)	66 (26.7)	40 (20.2)	
higher middle	681 (25.0)	19 (22.7)	66 (25.6)	53 (31.0)	
highest	701 (26.2)	27 (26.6)	55 (23.7)	48 (24.8)	
**Residence**					
Rural	1088 (32.9)	34 (26.8)	109 (34.5)	71 (28.7)	0.533
Urban	1611 (67.1)	64 (73.2)	136 (65.5)	115 (71.3)	
25(OH)D, nmole/L	52.85 ± 0.65	51.63 ± 2.73	52.45 ± 1.45	52.78 ± 1.98	0.967
PTH, pg/ mL	65.89 ± 0.85	64.69 ± 2.71	63.05 ± 1.68	66.51 ± 2.22	0.418
Age of TB diagnosis, years	—	29.7 ± 1.5	—	34.3 ± 1.3	0.022
Timing of TB diagnosis, years ago	—	30.7 ± 1.3	—	30.4 ± 1.3	0.884

Data are presented as frequency (weighted %) or means ± standard error. *Exercise was defined as engaging in moderate or vigorous exercise on a regular basis (at least three times per week, 20 min each time). ^†^Alcohol drinking was defined as more than once every month in the last year. BMI, body mass index; CXR, chest x-ray; Hx, history of physician diagnosis; 25(OH)D, 25-hydroxyvitamin D; PTH, parathyroid hormone; TB, tuberculosis; Hx (+)/CXR (−), subjects with a history of TB without TB scars on CXR; Hx (−)/CXR (+), subjects with TB scars on CXR without a history of TB; Hx (+)/CXR (+), subjects having both a history of TB and TB scars on CXR.

### Body composition values and BMD

Appendicular skeletal muscle mass (ASM) (*P* < 0.001, *P* for trend <0.001) and appendicular skeletal muscle mass index (ASMI) (*P* < 0.001, *P* for trend <0.001) tended to be lower in the following order: those with no evidence of TB or only a history of TB> those with only TB scars on CXR> those with both a history and TB scars on CXR. The proportion of subjects with presarcopenia and sarcopenia were higher (*P* < 0.001) in subjects with pulmonary TB scars on CXR compared to that of subjects without pulmonary TB scars on CXR. The proportions of sarcopenia (<2 standard deviation [SD]) for subjects without evidence of TB, with only a history of TB, with only TB scars on CXR, and with both a history and TB scars on CXR were 3.1%, 6.6%, 11.0%, and 14.5%, respectively (Table 2).

**Table 2:** Body composition values and BMD of study participants (n = 3,228).

	No evidence of pulmonary TB	TB survivors			*P*	*P* for trend
		Hx (+)/CXR (−)	Hx (−)/CXR (+)	Hx (+)/CXR (+)		
	n = 2,699	n = 98	n = 245	n = 186		
ASM, kg	21.26 ± 0.08	21.80 ± 0.46	19.70 ± 0.25	19.43 ± 0.27	<0.001	<0.001
ASMI, kg/m^2^	7.58 ± 0.02	7.63 ± 0.13	7.14 ± 0.08	6.96 ± 0.08	<0.001	<0.001
ASM/ Weight	31.56 ± 0.09	32.11 ± 0.31	31.74 ± 0.21	31.65 ± 0.23	0.278	0.314
Presarcopenia	556 (18.8)	24 (18.3)	86 (35.3)	69 (36.8)	<0.001	
Sarcopenia	101 (3.1)	7 (6.6)	28 (11.0)	21 (14.5)		
Total fat mass, kg	15.31 ± 0.15	14.45 ± 0.54	13.40 ± 0.37	13.60 ± 0.41	<0.001	<0.001
FMI, kg/m^2^	5.46 ± 0.05	5.07 ± 0.19	4.86 ± 0.13	4.87 ± 0.14	<0.001	<0.001
% body fat	22.52 ± 0.17	21.29 ± 0.62	21.23 ± 0.43	21.82 ± 0.50	0.004	0.004
Femur T	0.05 ± 0.02	0.15 ± 0.10	−0.49 ± 0.06	−0.53 ± 0.08	<0.001	<0.001
Femur neck T	−0.64 ± 0.02	−0.55 ± 0.13	−1.16 ± 0.06	−1.23 ± 0.08	<0.001	<0.001
Lumbar T	−0.59 ± 0.03	−0.52 ± 0.12	−0.97 ± 0.08	−1.11 ± 0.12	<0.001	<0.001
Osteopenia	1251 (44.3)	45 (38.1)	132 (51.8)	105 (58.6)	<0.001	
Osteoporosis	187 (5.9)	7 (6.8)	38 (13.5)	27 (14.6)		
Low BMD	1438 (50.3)	52 (44.9)	170 (65.3)	132 (73.2)	<0.001	

Data are presented as frequency (weighted %) or means ± standard error. ASM, appendicular skeletal mass; ASMI, appendicular skeletal mass index by height squared; BMD, bone mineral density; CXR, chest x-ray; FMI, fat mass index; Hx, history of physician diagnosis; TB, tuberculosis; Hx (+)/CXR (−), subjects with a history of TB without TB scars on CXR; Hx (−)/CXR (+), subjects with TB scars on CXR without a history of TB; Hx (+)/CXR (+), subjects having both a history of TB and TB scars on CXR.

**Figure 2:**
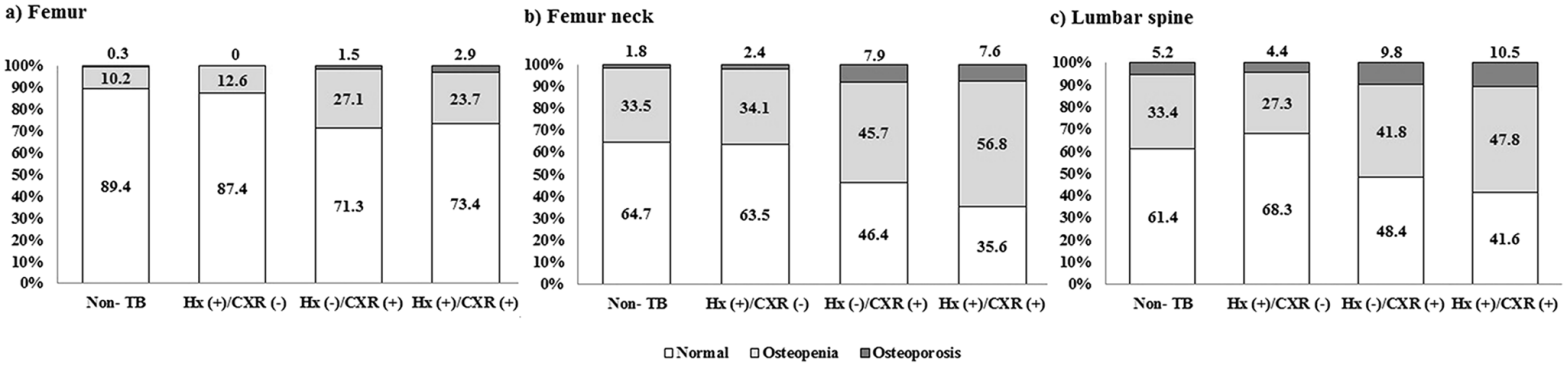
Proportion of subjects with osteopenia/osteoporosis among those with different evidence of prior TB. TB, tuberculosis; Hx (+)/CXR (−), subjects with a history of TB without TB scars on CXR; Hx (−)/CXR (+), subjects with TB scars on CXR without a history of TB; Hx (+)/CXR (+), subjects having both a history of TB and TB scars on CXR.

T scores of femur (*P* < 0.001, *P* for trend <0.001), femur neck (*P* < 0.001, *P* for trend <0.001), and lumbar spine (*P* < 0.001, *P* for trend <0.001) were lower in the following order: those with no evidence of TB or only a history of TB> those with only TB scars on CXR> those with both a history and TB scars on CXR. Proportions of osteoporosis for subjects without evidence of TB, with only a history of TB, with only TB scars on CXR, and with both a history and TB scars on CXR were 5.9%, 6.8%, 13.5%, and 14.6%, respectively (Table 2). Proportions of osteopenia/osteoporosis at femur neck and lumbar spine were higher in subjects with pulmonary TB scars on CXR compared to those of subjects without pulmonary TB scars on CXR (Fig. 2; *P* < 0.001 and *P* < 0.001, respectively).

### Risk of sarcopenia among TB survivors

After adjusting for age, height, smoking, alcohol, physical activity, serum 25-hydroxyvitamin D (25[OH]D) level, parathyroid hormone (PTH) level, education, and fat mass index (FMI), adjusted odds ratio (OR) for sarcopenia (ASMI <1 SD and ASMI <2 SD) tended to increase for those with only a history of TB (OR 1.16, 95% confidence interval [CI] 0.69–1.94; OR 2.23, 95% CI 0.78–6.41, respectively), only TB scars on CXR (OR 1.90, 95% CI 1.33–2.69; OR: 2.05, 95% CI 1.05–4.00, respectively), and those with both a history and TB scars on CXR (OR 2.64, 95% CI 1.71–4.08; OR 3.44, 95% CI 1.79–6.68, respectively) (both *P* for trend <0.001) (Table 3).

**Table 3:** Odds ratios (OR) of sarcopenia among TB survivors by evidence of prior pulmonary TB (n = 3,228).

	Sarcopenia (<1 SD)		P for trend	Sarcopenia (<2 SD)		*P* for trend
	OR (95% CI)	*P*		OR (95% CI)	*P*	
**Crude**						
No evidence of pulmonary TB	1			1		
Hx (+)/CXR (−)	1.18 (0.71–1.97)	0.519	<0.001	2.24 (0.83–6.00)	0.110	<0.0.001
Hx (−)/CXR (+)	3.08 (2.18–4.35)	<0.001		3.89 (2.12–7.13)	<0.0001	
Hx (+)/CXR (+)	3.75 (2.51–5.60)	<0.001		5.33 (2.96–9.61)	<0.0001	
**Model 1**						
No evidence of pulmonary TB	1			1		
Hx (+)/CXR (−)	1.20 (0.72–1.99)	0.489	<0.0001	2.31 (0.84–6.40)	0.107	<0.0.001
Hx (−)/CXR (+)	2.26 (1.58–3.21)	<0.0.001		2.70 (1.42–5.15)	0.003	
Hx (+)/CXR (+)	3.04 (2.01–4.60)	<0.0.001		4.17 (2.34–7.44)	<0.0.001	
**Model 2**						
No evidence of pulmonary TB	1			1		
Hx (+)/CXR (−)	1.33 (0.81–2.18)	0.259	<0.0.001	2.71 (0.96–7.66)	0.060	<0.0.001
Hx (−)/CXR (+)	2.17 (1.53–3.08)	<0.0.001		2.54 (1.36–4.72)	0.004	
Hx (+)/CXR (+)	3.06 (2.00–4.69)	<0.0.001		4.32 (2.35–7.93)	<0.0.001	
**Model 3**						
No evidence of pulmonary TB	1			1		
Hx (+)/CXR (−)	1.16 (0.69–1.94)	0.585	<0.001	2.23 (0.78–6.41)	0.135	<0.0.001
Hx (−)/CXR (+)	1.90 (1.33–2.69)	<0.001		2.05 (1.05–4.00)	0.037	
Hx (+)/CXR (+)	2.64 (1.71–4.08)	<0.0.001		3.44 (1.79–6.62)	<0.001	

Model 1: adjusted for age and height. Model 2: as Model 1, with additional adjustment for smoking, alcohol, activity, 25(OH)D, PTH, and education. Model 3: as Model 2, with additional adjustment for FMI. CXR, chest x-ray; Hx, history of physician diagnosis; TB, tuberculosis; Hx (+)/CXR (−), subjects with a history of TB without TB scars on CXR; Hx (−)/CXR (+), subjects with TB scars on CXR without a history of TB; Hx (+)/CXR (+), subjects having both a history of TB and TB scars on CXR.

### Risk of low BMD and osteoporosis among TB survivors

**Figure 3:**
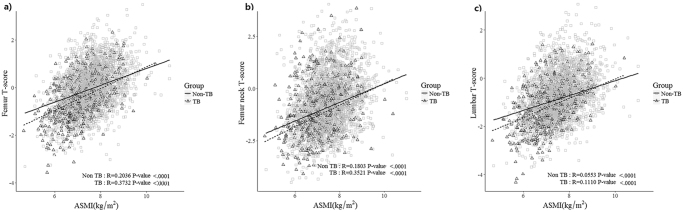
Scatter plot analysis of ASMI with T scores at femur (**a**), femur neck (**b**), and lumbar spine (**c**) using age adjusted linear regression. The positive association was more prominent in TB survivors compared to that in subjects without evidence of TB at femur, femur neck, and lumbar spine: at femur, R = 0.3732, *P < *0.001 vs. R = 0.2036, *P* < 0.001; femur neck, R = 0.3521, *P* < 0.001 vs. R = 0.1803, *P* < 0.001; and lumbar spine, R = 0.1110, *P* < 0.001 vs. R = 0.0553, *P* < 0.001. ASMI, appendicular skeletal muscle mass index; TB, tuberculosis.

After adjusting for the aforementioned variables, the adjusted OR for low BMD and osteoporosis tended to increase for those with only a history of TB (OR 0.79, 95% CI 0.48–1.30; OR 1.13, 95% CI 0.44–2.90, respectively), those with only TB scars on CXR (OR 1.26, 95% CI 0.89–1.78; OR 1.37, 95% CI 0.86–2.18, respectively), and those with both a history and TB scars on CXR (OR 2.14, 95% CI 1.38–3.31; OR 1.75, 95% CI 1.04–2.95, respectively) (*P* for trend <0.001, *P* for trend = 0.028, respectively) (Table 4). ASMI values were positively correlated with T scores at femur, femur neck, and lumbar spine after adjustment for age. This positive association was more prominent in TB survivors compared to that in subjects without evidence of TB at femur, femur neck, or lumbar spine: at femur, R = 0.3732, *P < *0.001 vs. R = 0.2036, *P* < 0.001; at femur neck, R = 0.3521, *P* < 0.001 vs. R = 0.1803, *P* < 0.001; at lumbar spine, R = 0.1110, *P* < 0.001 vs. R = 0.0553, *P* < 0.001 (Fig. 3).

**Table 4:** Odds ratios (OR) of low BMD and osteoporosis among TB survivors by evidence of prior pulmonary TB (n = 3,228).

	Low BMD			Osteoporosis		*P* for trend
	OR (95% CI)	*P*	*P* for trend	OR (95% CI)	*P*	
**Crude**						
No evidence of pulmonary TB	1			1		
Hx (+)/CXR (−)	0.81 (0.49–1.32)	0.393	<0.0.001	1.16 (0.46–2.92)	0.755	<0.0.001
Hx (−)/CXR (+)	1.86 (1.35–2.57)	<0.001		2.47 (1.60–3.81)	<0.0.001	
Hx (+)/CXR (+)	2.71 (1.79–4.09)	<0.0.001		2.72 (1.63–4.53)	<0.001	
**Model 1**						
No evidence of pulmonary TB	1			1		
Hx (+)/CXR (−)	0.84 (0.52–1.37)	0.485	<0.0.001	1.22 (0.49–3.07)	0.670	0.001
Hx (−)/CXR (+)	1.44 (1.03–2.03)	0.036		1.69 (1.08–2.65)	0.022	
Hx (+)/CXR (+)	2.34 (1.52–3.61)	<0.001		2.13 (1.25–3.63)	0.005	
**Model 2**						
No evidence of pulmonary TB	1			1		
Hx (+)/CXR (−)	0.86 (0.52–1.40)	0.539	<0.0.001	1.34 (0.55–3.31)	0.520	0.001
Hx (−)/CXR (+)	1.38 (0.98–1.95)	0.069		1.66 (1.05–2.60)	0.029	
Hx (+)/CXR (+)	2.34 (1.52–3.61)	<0.001		2.15 (1.27–3.62)	0.004	
**Model 3**						
No evidence of pulmonary TB	1			1		
Hx (+)/CXR (−)	0.79 (0.48–1.30)	0.358	<0.001	1.13 (0.44–2.90)	0.803	0.028
Hx (−)/CXR (+)	1.26 (0.89–1.78)	0.194		1.37 (0.86–2.18)	0.190	
Hx (+)/CXR (+)	2.14 (1.38–3.31)	<0.001		1.75 (1.04–2.95)	0.036	

Model 1: adjusted for age and height. Model 2: as Model 1, with additional adjustment for smoking, alcohol, activity, 25(OH)D, PTH, and education. Model 3: as Model 2, with additional adjustment for FMI. BMD, bone mineral density; CXR, chest x-ray; Hx, history of physician diagnosis; TB, tuberculosis; Hx (+)/CXR (−), subjects with a history of TB without TB scars on CXR; Hx (−)/CXR (+), subjects with TB scars on CXR without a history of TB; Hx (+)/CXR (+), subjects having both a history of TB and TB scars on CXR.

**Figure 4:**
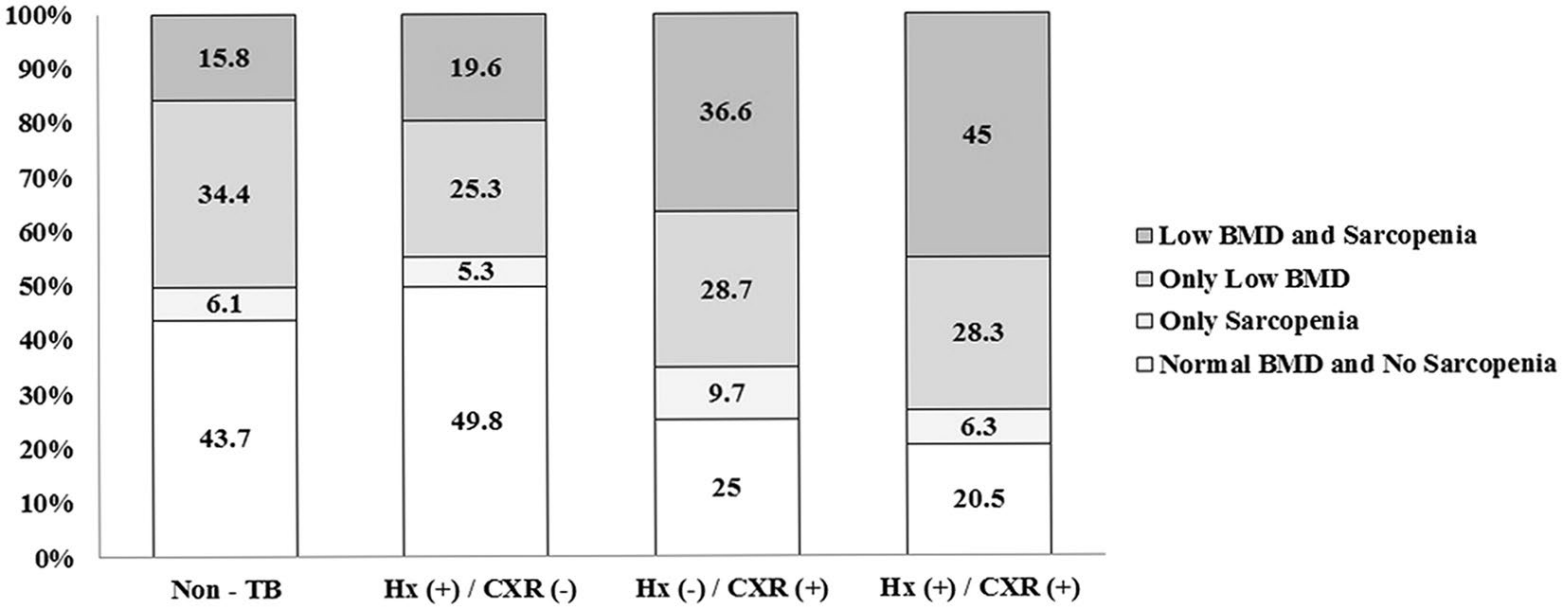
Proportion of those with low BMD or sarcopenia (ASMI <1 SD) among subjects with different evidence of prior TB. ASMI, appendicular skeletal muscle mass index; BMD, bone mineral density; CXR, chest x-ray; Hx, history of physician diagnosis; TB, tuberculosis; Hx (+)/CXR (−), subjects with a history of TB without TB scars on CXR; Hx (−)/CXR (+), subjects with TB scars on CXR without a history of TB; Hx (+)/CXR (+), subjects having both a history of TB and TB scars on CXR.

Proportions of subjects with overlapping low BMD and sarcopenia (<1 SD) for those without evidence of TB, those with only a history of TB, those with only TB scars on CXR, and those with both a history of TB and TB scars on CXR were 15.8%, 19.6%, 36.6%, and 45.0%, respectively (Fig. 4).

## Discussion

In this national representative Korean population study of males who were 50 years of age or older, subjects with both a medical history and TB scars on their CXR had increased risk of sarcopenia (OR 3.44, 95% CI 1.79–6.68) and osteoporosis (OR 1.75, 95% CI 1.04–2.95) after adjusting for confounding factors. Having TB scars on CXR without medical history of TB was an independent risk factor of sarcopenia (OR 2.05, 95% CI 1.05–4.00). However, it was not a risk factor of osteoporosis. TB survivors without TB scars on CXR did not have an increased risk of sarcopenia or osteoporosis. These results differ from the impact of pulmonary TB on respiratory function. It has been reported that subjects having only a history of pulmonary TB without TB scars on CXR have lower pulmonary function and higher proportion of air flow obstruction than subjects without evidence of TB^14^.

Age-related osteoporosis in men is typically seen in those over 70 years of age. Male osteoporosis remains underdiagnosed and undertreated^15^. However, about 25% of men over 50 years of age will develop at least one osteoporosis-related fracture in their lifetime. Compared to women, men have a higher rate of mortality within one year after hip fracture^16^. BMD values are only surrogate markers for fracture risk. They do not fully predict future fractures^17^. Falls and poor neuromuscular function are risk factors of osteoporosis-related fractures in men independent of BMD^18,19^. Sarcopenia is an important risk for falling^20,21^ and physical disability^22,23^. It is also considered a risk factor for low BMD^24–26^. Men having both low BMD and sarcopenia reportedly have a four times higher risk of fractures than men with normal BMD without sarcopenia^27^. In the present study, the proportion of those with overlapping low BMD and sarcopenia was 45.0% in subjects with both a medical history and TB scars on CXR. These subjects might have increased risk of osteoporosis-related fracture. The present findings are consistent with results of recent retrospective cohort studies in Taiwan. In these studies, frequently hospitalized patients with pulmonary TB have higher rates of new onset osteoporosis^28^ and osteoporotic fracture^28,29^ than matched cohorts. Past history of active TB has been found to be an independent risk factor for incident osteoporosis and osteoporotic fracture^28^.

Pathogenesis of sarcopenia or osteoporosis in TB survivors is currently unknown. *Mycobacterium tuberculosis* infection can lead to a wide range of clinical manifestations ranging from asymptomatic infection to death. Only 10% of individuals exposed to *M. tuberculosis* will develop active disease, indicating that host immune response is an important factor. As a marker of under-nutrition, low BMI is the most obvious host factor for TB infection. It can increase the risk of active infection^3^, disease severity, clinical outcomes^4–7^, and death from pulmonary TB^8^. Low BMI is also a well-known risk factor for sarcopenia and low BMD. Low BMI may be an innate host trait associated with TB infection. It might increase the risk of sarcopenia and low BMD later in life. TB is a wasting disease. Most patients gain weight during treatment. However, short-term prospective studies on changes of body composition after treatment suggest that TB can lead to permanent loss of lean tissue and fat mass^11–13^. The destructive nature of pulmonary TB may induce chronic lung impairment even if TB treatment is effective^30,31^. In one study, subjects with both medical history and TB lesions on CXR have a 4.5-times higher risk for chronic obstructive pulmonary disease (COPD) and 2.7-times increased risk for restriction dysfunction compared to subjects without evidence of TB^14^. Osteoporosis and sarcopenia are frequently found in patients with COPD^32^. Physical inactivity, increased levels of systemic inflammation, hypoxia, poor nutrition, and use of corticosteroids have been implicated as major contributing factors^33^. Vitamin D deficiency is a risk factor of active TB and a sustained risk factor for TB recurrence even after recovery from active TB infection^34,35^. Vitamin D deficiency and compensatory rise in PTH may influence the pathogenesis of increased osteoporosis risk in patients with TB. In this study, serum level of 25(OH)D and PTH were not significantly different between TB survivors and subjects without evidence of TB. Information concerning supplementary vitamin D intake of participants was unavailable in the Korea National Health and Nutrition Examination Survey (KNHANES). A prospective study is needed to confirm this finding.

The present study does have several limitations. First, this was a cross-sectional study. It was impossible to determine a causal relationship. Second, sarcopenia was defined solely by low skeletal muscle mass. Muscle strength or physical performance was not considered in sarcopenia evaluation^36^. Third, information on the use of drugs was unavailable, making it difficult to estimate the possible influence of drugs on BMD. Fourth, TB is a deadly infectious disease with increased mortality. Therefore, the risk of sarcopenia and osteoporosis could be underestimated in TB survivors. Fifth, the prevalence of pulmonary TB survivors could lead to misclassification because it was based on participant’ report of their physician’s diagnosis or TB scars on CXR. However, the Korean national TB surveillance system has been ongoing since 1965. It is likely that physician’s diagnosis is fairly accurate compared to that in other countries. Further prospective and longitudinal studies where the diagnosis of TB is based on objective criteria are needed to confirm our findings.

Despite adequate anti-TB treatment, TB survivors had significant loss of predicted longevity compared to a similar group without history of active TB^37^. Post-TB respiratory impairment is a well-known cause of increased morbidity and mortality. Our findings suggest that sarcopenia and osteoporosis are also causes of higher morbidity and mortality burdens following TB.

In this study, TB survivors without TB scars did not have increased risk of sarcopenia or low BMD. Early detection and complete cure of pulmonary TB could reduce the risk of musculoskeletal sequelae in TB survivors. During and after TB treatment, efforts geared toward prevention of sarcopenia and osteoporosis are needed. For TB survivors with risk factors, early detection and treatment for sarcopenia and osteoporosis are also warranted to prevent the occurrence of osteoporotic fractures and fragility.

In conclusion, sarcopenia and low BMD are prevalent in pulmonary TB survivors with TB scars on CXR. Medical history of TB with TB scars on CXR is an independent risk factor for sarcopenia and osteoporosis.

## Materials and Methods

### Study population and data collection

This study was based on data obtained from the KNHANES between 2008 and 2011. KNHANES is a population-based, cross-sectional, and nationally representative survey designed to examine the health and nutritional status of civilian non-institutionalized population of the Republic of Korea. KNHANES followed a multi-stage cluster probability sampling design to ensure independent and homogenous sampling for each year as well as nationally representative sampling. Among 5,872 male subjects aged 50 years or older, a total of 3,228 participants who underwent CXR and DEXA were identified. Subjects who had active TB (n = 29), who did not receive DEXA (n = 1,196), who had no CXR data (n = 1,186), non-responders to health questionnaires (n = 76), and those who had no data for BMI or PTH level (n = 157) were excluded (Fig. 1). The Korea Centers for Disease Control and Prevention (KCDC) obtained written and informed consent from all participants. All study protocols were approved by KCDC Institutional Review Board (Approval no. 2008-04EXP-01-C, 2009-01CON-03-2C, 2010-02CON-21-C, and 2011-02CON-06-C). All methods were carried out in accordance with the approved guidelines and regulations.

### CXR and definition of TB survivors

Since 2008, the KNHANES has included a TB section to their health survey by performing population-wide screening with CXR to estimate the national prevalence of pulmonary TB. CXR images were taken with DigiRAD-PG (Sitec Medical Co., Ltd, Kimpo-si, Gyeonggi-do, Korea) installed on an examination vehicle. Two radiologists independently interpreted CXR results for the presence of lung disease. Individual readings were compared weekly and CXR results demonstrating TB related lesions were re-interpreted by six radiology specialists to confirm the results.

TB survivors were defined as those with a self-reported history of physician-diagnosed TB or TB lesions on CXR. They were categorized into the following TB subgroups: 1) those with history of TB but had no TB scars on CXR, 2) those had TB scars on CXR without a history of TB, and 3) those had both a history of TB and TB scars on CXR.

### DEXA and definitions of sarcopenia and low BMD

Body composition was analyzed with DEXA using a Discovery fan beam densitometer (Hologic Inc., Bedford, MA, USA). ASM was calculated as the sum of skeletal muscle in arms and legs. ASMI was defined as ASM divided by the square of height. Sarcopenia and presarcopenia were defined according to the presence of ASMI values that were <2 SD and between 1 and 2 SD, respectively, below the mean value of a young male reference group aged 20–39 years. Calculated ASMI cut-off values for sarcopenia were 6.96 kg/m^2^ for 1 SD and 6.06 kg/m^2^ for 2 SD, respectively. Subjects with presarcopenia or sarcopenia were collectively termed ‘sarcopenia <1 SD’. FMI was calculated as the sum of whole body fat divided by the square of height.

Osteoporosis, osteopenia, and normal BMD were identified according to the lowest T-score of femur, femur neck, and lumbar spine. They were defined according to the World Health Organization criteria: osteoporosis, T-score ≤−2.5; osteopenia, T-score between −2.5 and −1; and normal BMD. T-score >−1 ^38^. Subjects with either osteoporosis or osteopenia were collectively termed ‘low BMD’.

### Lifestyle and biochemical variables

Demographics, personal medical history, and lifestyle behaviors such as cigarette smoking, alcohol consumption, and physical activity of participants were collected from standardized health questionnaires. Smoking was defined as lifetime smoking of more than 100 cigarettes, including both current and past smoking. Regular exercise was defined as engaging in moderate or vigorous exercise on a regular basis (at least three times per week, 20 min each time). Regular alcohol dinking was defined as more than once every month in the last year. Household income was categorized according to quartile of total income of each member in the household.

Collected blood samples were analyzed within 24 hours after transportation in a central laboratory. Level of 25(OH)D was measured by radioimmunoassay (DiaSorin, Stillwater, MN) using a Wizard model 1470 gamma counter (PerkinElmer, Finland). Plasma PTH was measured by N-tact PTH assay using LIAISON chemiluminescence immunoassay system (DiaSorin, Stillwater, MN, USA).

### Statistical analyses

To produce an unbiased national estimate, a sample weight was assigned for participating individuals in order to represent the Korean population. Sampling weights were constructed to account for the complex survey design, survey non-response, and post-stratification. Continuous variables were expressed as means with standard errors while categorical variables were presented as cases per category and frequency of responses. Baseline characteristics, body composition values, and BMD of participants based on historical or radiographic evidence of pulmonary TB were analyzed by analysis of variance (ANOVA) or Chi-square test. The association of different groups for sarcopenia or low BMD was modeled by logistic regression after adjusting for age, height, smoking, alcohol, physical activity, 25(OH) D level, PTH level, education, and FMI. *P* for trend was calculated by assigning the mean of body composition values and odds ratio of distinct TB evidence groups as continuous variables. Age adjusted linear regression analysis and scatter plot analysis were performed between ASMI and T scores at femur, femur neck, and lumbar spine. All statistical analyses were performed with SAS version 9.4 (SAS Institute, Cary, NC, USA) and a value of *P < *0.05 was considered statistical significance.

### Data Availability

This study was based on data obtained from KNHANES between 2008 and 2011. The datasets are available from the official website of KNHANES: https://knhanes.cdc.go.kr/knhanes/main.do.English-language information is available via. https://knhanes.cdc.go.kr/knhanes/eng/index.do.
